# Differential cellular stiffness across tissues that contribute to *Xenopus* neural tube closure

**DOI:** 10.1111/dgd.12936

**Published:** 2024-06-26

**Authors:** Makoto Suzuki, Naoko Yasue, Naoto Ueno

**Affiliations:** ^1^ Amphibian Research Center, Graduate School of Integrated Sciences for Life Hiroshima University Hiroshima Japan; ^2^ Division of Morphogenesis, National Institute for Basic Biology National Institutes of Natural Sciences Aichi Japan; ^3^ Basic Biology Program the Graduate University of Advanced Studies Aichi Japan

**Keywords:** Actomyosin, atomic force microscopy, neural tube closure, *Xenopus*, Young's modulus

## Abstract

During the formation of the neural tube, the primordium of the vertebrate central nervous system, the actomyosin activity of cells in different regions drives neural plate bending. However, how the stiffness of the neural plate and surrounding tissues is regulated and mechanically influences neural plate bending has not been elucidated. Here, we used atomic force microscopy to reveal the relationship between the stiffness of the neural plate and the mesoderm during *Xenopus* neural tube formation. Measurements with intact embryos revealed that the stiffness of the neural plate was consistently higher compared with the non‐neural ectoderm and that it increased in an actomyosin activity‐dependent manner during neural plate bending. Interestingly, measurements of isolated tissue explants also revealed that the relationship between the stiffness of the apical and basal sides of the neural plate was reversed during bending and that the stiffness of the mesoderm was lower than that of the basal side of the neural plate. The experimental elevation of mesoderm stiffness delayed neural plate bending, suggesting that low mesoderm stiffness mechanically supports neural tube closure. This study provides an example of mechanical interactions between tissues during large‐scale morphogenetic movements.

## Graphical abstract

In this study, Suzuki et al. investigated the stiffness of the neural plate and surrounding tissues in *Xenopus* neural tube formation using atomic force microscopy and observed that the relationship between the stiffness of the apical and basal sides of the neural plate was reversed during neural plate bending and that the stiffness of the mesoderm was lower than that of the basal side of the neural plate. Furthermore, the experimental elevation of mesoderm stiffness delayed neural plate bending, suggesting that low mesoderm stiffness mechanically supports neural tube formation.

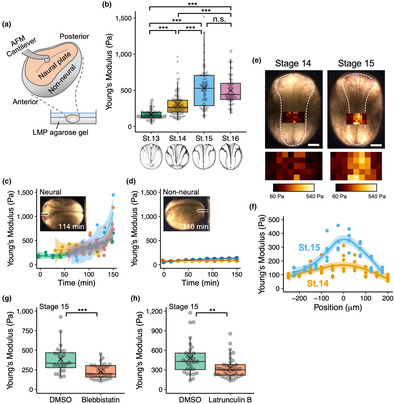

## 1 INTRODUCTION

The neural tube, the primordium of the central nervous system, is formed as a tube‐like structure along the rostral–caudal axis on the dorsal surface of vertebrate embryos. During neural tube formation, the lateral edges of the neural plate, a flat cell sheet composed of neural progenitor cells, elevate toward the midline and then fuse at the most dorsal part (Colas & Schoenwolf, 2001). This process is one of the most extensive morphogenetic movements in vertebrate embryogenesis and is known as neural tube closure (NTC). Failure of the NTC causes neural tube defects (NTDs), which are common and severe birth defects in humans (Copp et al., 2003; Wilde et al., 2014).

NTC is mechanically driven by a spatial bias in actomyosin activity on the cell surface of the neural plate. In neural progenitor cells, F‐actin accumulates on the apical side, where it is linked to cell–cell adhesion via underlying proteins such as α‐catenin (Martin & Goldstein, 2014; Nandadasa et al., 2009; Sawyer et al., 2010; Suzuki et al., 2012; Suzuki et al., 2017). F‐actin cooperates with non‐muscle myosin activated by Shroom3 and Rho‐kinase, which act downstream of the non‐canonical Wnt signaling pathway, to generate higher contractile activity levels on the apical side relative to the basolateral side (Haigo et al., 2003; Martin & Goldstein, 2014; McGreevy et al., 2015; Nandadasa et al., 2009; Nishimura et al., 2012; Nishimura & Takeichi, 2008; Sawyer et al., 2010; Suzuki et al., 2012). This bias in contractile activity within the cell causes morphogenesis at the cellular level within the neural plate, known as apical constriction, which reduces the apical cell surface area and contributes to the inward bending of the neural plate (Inoue et al., 2016).

Cellular stiffness is a mechanical property of tissues, which are aggregates of cells. In early embryos, stiffness is directly regulated by the distribution and magnitude of the actomyosin activity (Barriga et al., 2018; Zhou et al., 2009; Zhou et al., 2010). Therefore, the cellular stiffness within the neural plate may change spatiotemporally during NTC. A recent analysis using a non‐invasive Brillouin microscopy technique suggested that the mechanical properties of the neural plate of living chicken embryos increased during NTC (Handler et al., 2023). However, this has not been experimentally validated by direct measurements; the actual stiffness of the neural plate and its changes during NTC remain unclarified. In addition, the balance of stiffness between the neural plate and its surrounding tissues, such as the non‐neural ectoderm and mesoderm adjacent to the lateral and ventral sides of the neural plate, respectively, and its significance, remain unknown. In this study, we used *Xenopus laevis* embryos as a model to address the above questions by analysis using atomic force microscopy.

## 2 MATERIALS AND METHODS

### 2.1 *Xenopus* embryo manipulation and microinjection

Experiments with *X. laevis* embryos were performed as previously described (Suzuki et al., 2017). Briefly, embryos were raised in our laboratory by in vitro fertilization (Sive et al., 2000) and cultured in 0.3 × Marc's modified Ringer's medium (0.3 × MMR: 30 mM NaCl, 0.6 mM KCl, 0.3 mM MgCl_2_, 0.6 mM CaCl_2_, and 1.5 mM HEPES [pH 7.4]) until the appropriate stage for analysis (Nieuwkoop & Faber, 1967).

To measure the stiffness of the intact embryo, devitellinized embryos were embedded in a 1% low‐melting‐point agarose solution in a 60‐mm plastic Petri dish. After the agarose drop was set, the dish was filled with 0.3 × MMR. Excess agarose gel covering the neural plate was removed using micro‐dissecting forceps. To reduce actomyosin activity levels, embryos were treated with 50 μM blebbistatin (#13013; Cayman) for 3–4 h or 0.5 μM latrunculin B (BML‐T110; Enzo) for 10 min before being embedded.

To measure the stiffness of tissue explants, devitellinized embryos with incisions made around the dorsal tissue were treated with 1 mg/mL Collagenase A (#10103578001; Roche) in 1 × Steinberg's solution (58 mM NaCl, 0.67 mM KCl, 0.34 mM Ca(NO_3_)_2_, 0.83 mM MgSO_4_, and 0.46 mM Tris–HCl [pH 7.4]) for 15–20 min. The neural and dorsal mesodermal tissues were then cut out using a tungsten needle and an eyebrow knife in a modified Danilchik's for Amy (DFA) solution (53 mM NaCl, 5 mM Na_2_CO_3_, 4.5 mM potassium gluconate, 32 mM sodium gluconate, 1 mM CaCl_2_, 1 mM MgSO_4_, and 0.1% bovine serum albumin). The pH was adjusted to 8.3 with 1 M bicine before adding the bovine serum albumin. The excised explants were fixed on 60‐mm plastic Petri dishes filled with a modified DFA solution using pieces of thinly pulled glass capillaries and silicon grease.

To manipulate mesoderm stiffness experimentally, in vitro transcribed capped mRNAs or antisense morpholino‐oligonucleotides (MOs) were injected into two right dorso‐vegetal blastomeres (prospective mesoderm) of 16‐cell‐stage embryos. Enhanced green fluorescent protein (EGFP) mRNA was co‐injected as a tracer in all microinjection experiments. Capped mRNAs for EGFP, constitutively active *arhgef2*‐C55R (Zhou et al., 2010), and a constitutively active form of the myosin light chain (CA‐MLC) (Iwasaki et al., 2001) were synthesized using the mMESSAGE mMACHINE SP6 Transcription kit (AM1340; Thermo Fisher) and purified with the NICK Columns Sephadex G‐50 (#17085501; GE Healthcare). CA‐MLC was subcloned into the pCS2p + vector for in vitro transcription. MOs for the standard control (Std‐MO 5′‐CCTCTTACCTCAGTTACAATTTATA‐3′) and myosin light chain 9 (*myl9*‐MO 5′‐TGGCTTTTGTCTTCTTGCTGGACAT‐3′) (Shindo & Wallingford, 2014) were synthesized by GeneTools. The amounts of injected mRNAs and MOs per blastomere were as follows: EGFP, 50 pg; *arhgef2*‐C55R, 25 pg; CA‐MLC, 1 ng; Std‐MO and *myl9*‐MO, 2 ng. The injected embryos were cultured in 3% Ficoll/0.1 × Steinberg's solution to stage 9 and then cultured in 0.3 × MMR.

### 2.2 Atomic force microscopy measurements

**FIGURE 1:**
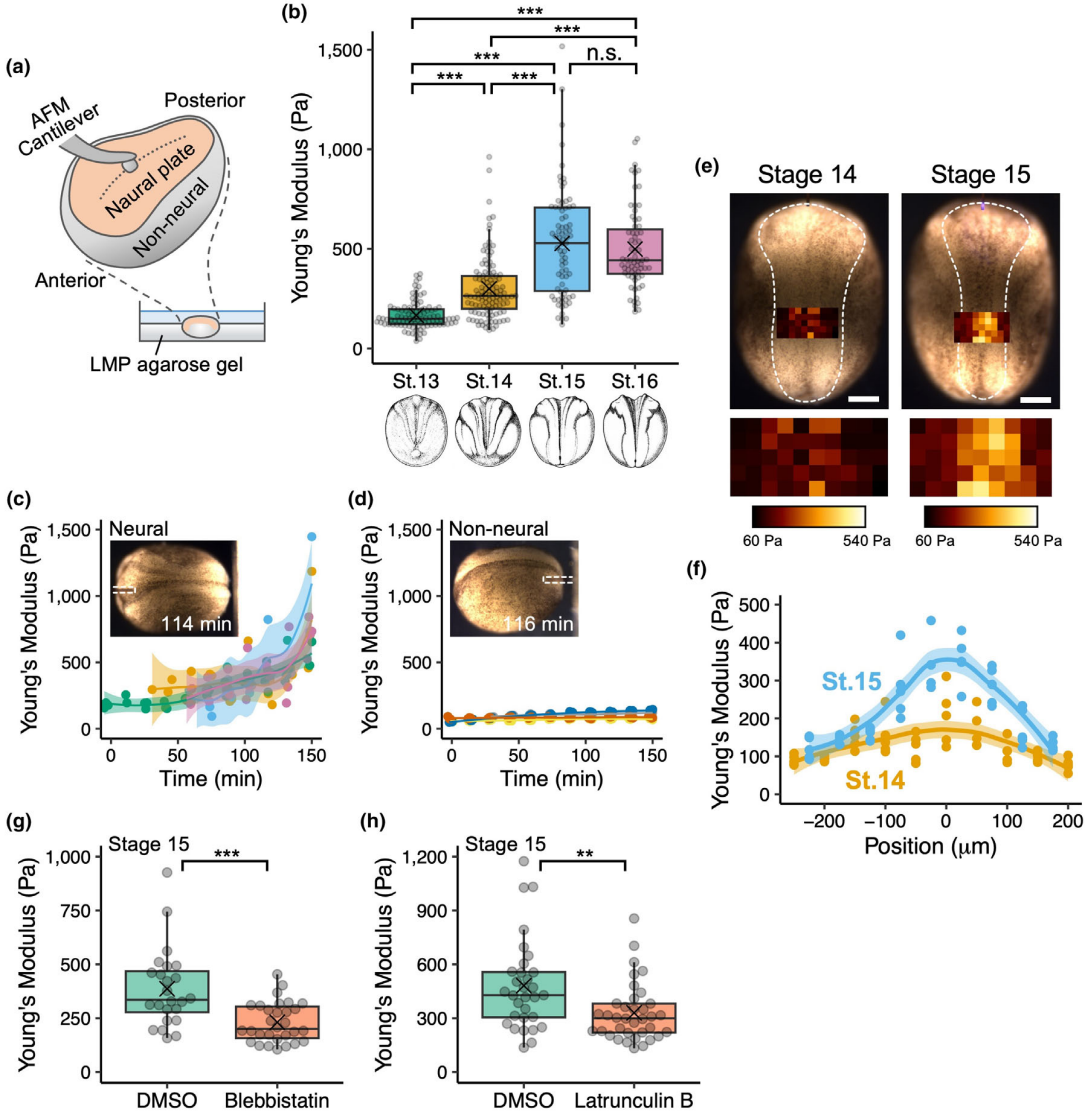
Elasticity of neural plates measured with atomic force microscopy (AFM). (a) Schematic of the AFM measurement. A devitellinized embryo is fixed on a plastic dish using a low‐melting‐point agarose gel. The surface of the neural plate (orange) is indented using a bead‐attached AFM cantilever. The dashed line indicates the midline. Indentations are made in the presumptive anterior spinal cord. (b) Stiffness at stages 13–16: *n* = 99 indentations, 31 embryos (stage 13); 114 indentations, 36 embryos (stage 14); 75 indentations, 27 embryos (stage 15); and 67 indentations, 24 embryos (stage 16). Stiffness increased from stage 13 to stage 15 (*p*_stage13–14_ = 4.3 × 10^−13^, *p*_stage14–15_ = 1.1 × 10^−9^, *p*_stage15–16_ = .66). (c, d) Stiffness of the neural plate (c) and non‐neural ectoderm (d), measured by repeated indentations of the same embryos (*n* = 4 each). The 0‐ and 150‐min marks correspond approximately to stages 13 and 16, respectively. The insets show representative images of stage 15 embryos during the measurements. Anterior is to the left. The dashed lines indicate the cantilevers at the rest position. (e) Two‐dimensional mapping of neural plate stiffness at stages 14 (left) and 15 (right). Anterior is to the top. Magnified views of the color‐coded stiffness map are shown at the bottom. Scale bars: 250 μm. (f) A plot of stiffness along the mediolateral axis at stages 14 (orange) and 15 (blue), shown in (e). Zero, negative, and positive values correspond to the midline, left, and right sides, respectively. (g, h) Stiffness of the neural plate treated with blebbistatin (g) and latrunculin B (h); *n* = 24 indentations, 9 embryos (DMSO in g); 30 indentations, 10 embryos (blebbistatin); 31 indentations, 10 embryos (DMSO in h); and 36 indentations, 12 embryos (latrunculin B). Both treatments reduced neural plate stiffness compared with DMSO controls (*p*_blebbistatin_ = 7.8 × 10^−5^, *p*_latrunculin B_ = 3.0 × 10^−3^). In (b), (g), and (h), the box and whiskers indicate the maximum, third quartile, median, first quartile, and minimum values of each group; data beyond the end of the whiskers (the upper limit of whiskers is 1.5 times the box length) are shown as outliers; a cross mark indicates the mean value. In (c), (d), and (f), the colored line and shaded areas indicate the locally estimated scattered smoothing regression and confidence interval of each embryo, respectively. *p*‐values were calculated using the two‐sided Wilcoxon rank‐sum test (**p < .01; ***p < .001; n.s., not significant). *p*‐values for pairwise comparisons were adjusted using Holm's method.

Atomic force microscopy (AFM) measurements were performed as described previously (Kinoshita et al., 2020; Koyama et al., 2022). Briefly, a JPK CellHesion 200 (Bruker) fitted with an x/y‐motorized stage and mounted on a macro zoom microscope (Axio Zoom.V16; Zeiss) was used. Customized AFM probes (Novascan) were prepared by attaching SiO_2_ beads (10‐μm diameter) to tipless rectangular silicon nitride cantilevers (210 μm long, 20 μm wide; nominal spring constant 0.02 N/m; MLCT‐B; Bruker). The measured spring constants were in the range of 0.015 to 0.019 N/m. In the repeated measurements of the same embryos (cf. Figure 1c, d), the probes were prepared by attaching borosilicate beads (20‐μm diameter) to tipless rectangular silicon cantilevers (350 μm long, 32.5 μm wide, 1 μm thick; nominal spring constant 0.03 N/m; MikroMasch). The measured spring constants were in the range of 0.059 to 0.067 N/m.

Force–indentation curves were acquired using the JPK data processing software (Bruker). The measurement conditions were as follows: set point, 5–13 nN; approach speed, 2 μm/s; and sampling rate, 500 Hz. The cell elasticity (Young's modulus) values on the tissue surface were calculated based on the Hertz model (Hertz, 1881) for indentation depths of 3 μm, which has been used to measure the mechanical properties of embryonic tissues (Barriga et al., 2018; Iwashita et al., 2014; Kinoshita et al., 2020; Koser et al., 2016). For the two‐dimensional mapping (cf. Figure 1e, f), force–indentation curves were acquired every 50 μm in a bidirectional raster scan, and the calculated elasticity values were mapped onto brightfield images using the JPK data processing software.

### 2.3 Fixation, staining, and imaging

To examine gross morphological defects, injected embryos were fixed for 2 h with modified MEMFA (0.1 M MOPS [pH 7.4], 2 mM EGTA, 1 mM MgSO_4_, 3.7% formaldehyde, 1% glutaraldehyde, and 0.1% Tween‐20) and observed with a macro zoom microscope (Axio Zoom.V16; Zeiss). For section imaging, embryos were fixed for 2 h with MEMFA and stained with Alexa Fluor 546 Phalloidin (A22283; Molecular Probes). Afterwards, 100‐μm thick transverse sections were prepared with a vibratome and observed with a confocal microscope (TCS SP8; Leica). Whole‐mount in situ hybridization was performed as previously described (Sive et al., 2000). The following plasmids from the *Xenopus* cDNA library (https://xenopus.nibb.ac.jp) were used for probe synthesis: *sox2.S* (XL039o24) and *actc1.L* (XL312h14ex) (Goda et al., 2009; Suzuki et al., 2010). The sequences of the plasmids were analyzed using the *Xenopus* genome database (http://viewer.shigen.info/xenopus/).

### 2.4 Image processing and statistical analysis

The acquired images were processed using the ZEN (Zeiss) and Fiji software (Schindelin et al., 2012), as previously described (Ishii et al., 2023). Data plots and statistical analyses were performed using the R software (R Core Team, Vienna, Austria) and Excel (Microsoft).

## 3 RESULTS AND DISCUSSION

### 3.1 Actomyosin‐dependent increase of elasticity in the neural plate

To investigate the mechanical properties of cells and tissues in the NTC, we took the approach to directly measure stiffness using AFM. This analysis calculates the elastic modulus of the cell and tissue surfaces from the amount of deformation of the probe with a known spring constant when the sample is indented (Barriga et al., 2018; Iwashita et al., 2014; Kinoshita et al., 2020; Koser et al., 2016). To perform indentation while observing *Xenopus* embryos that were less transparent, we constructed a system that combined an upright zoom microscope and AFM (Kinoshita et al., 2020; Koyama et al., 2022). Most of the tissue stiffness values of *Xenopus* embryos measured in this study were in the range of 50 to 1000 Pa, which is comparable to the values of other tissues measured in previous studies (Barriga et al., 2018; Hirano et al., 2022; Kinoshita et al., 2020; Koser et al., 2016).

First, we measured the stiffness of the neural plates of intact embryos. To establish the physical stability of the measurement surface required for AFM measurements, devitellinized embryos were placed in a dish with a low‐melting‐point agarose gel (Figure 1a). The neural plate surfaces in the presumptive anterior spinal cord region of these embryos were indented at stages 13–16, which corresponded to the period from the initiation of neural fold elevation to just before the completion of NTC, and elasticity was calculated. From this analysis, we observed that the elasticity of the neural plate increased from stages 13 to 15 (*p*_stage13–14_ < 10^−13^, *p*_stage14–15_ < 10^−8^, *p*_stage15–16_ = .66; Figure 1b). This observation is in good agreement with observations on the posterior neuroectoderm during gastrulation (Hirano et al., 2022). A similar tendency of increasing elasticity over time was detected in repeated indentations of the neural plate in the same embryos (Figure 1c, Movie S1). Measurements in the non‐neural ectoderm showed that its elasticity was consistently lower than that of the neural plate and did not increase during NTC (Figure 1d, Movie S2).

To investigate the elasticity of the lateral region of the neural plate, two‐dimensional indentations at stages 14 and 15 were performed using bidirectional raster scans. In both stages, the highest elasticity values were observed near the midline, gradually decreasing laterally (Figure 1e). Between stages 14 and 15, the lateral elasticity did not change significantly, whereas the elasticity near the midline increased (Figure 1f). This is consistent with the results described above (Figure 1b, c).

To verify the contribution of actomyosin activity, we measured the elasticity of embryos treated with blebbistatin, an inhibitor of non‐muscle myosin II, and latrunculin B, an inhibitor of F‐actin polymerization. The results showed that treatment with either inhibitor reduced the elasticity of the neural plate compared with that of the controls (*p*_blebbistatin_ < 10^−4^, *p*_latrunculin B_ = .0030; Figure 1g, h). These results suggest that the stiffness of the neural plate increases in an actomyosin‐dependent manner. A minimal increase in elasticity was observed in the non‐neural ectoderm (Figure 1d), suggesting that the significant change in elasticity of the neural plate is a phenomenon specific to extensive morphogenetic movements that do not occur in the surrounding tissues during the same period.

### 3.2 Relationship of elasticity between the apical and basal surfaces of the neural plate

**FIGURE 2:**
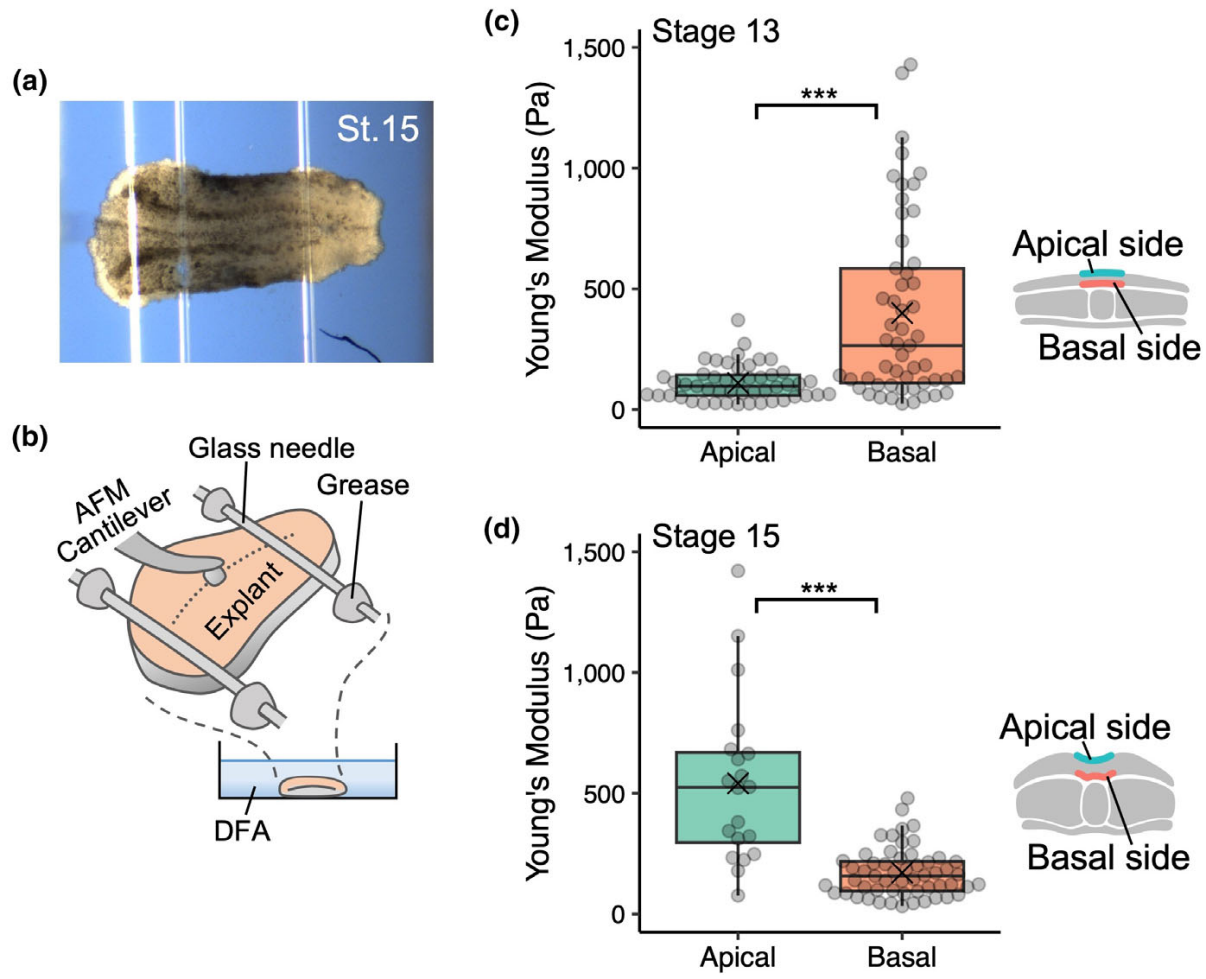
Elasticity of neural plate explants. (a, b) A representative image (a) and schematic (b) of atomic force microscopy (AFM) measurement. Anterior is to the left. An excised neural plate explant is fixed on a plastic dish using pieces of thinly pulled glass capillaries and silicon grease. The explant surface (orange) is indented using a bead‐attached AFM cantilever. The dashed line indicates the midline. (c, d) Stiffness of the apical and basal surfaces of the neural plate at stages 13 (c) and 15 (d); *n* = 49 indentations, 4 embryos (stage 13, apical); 54 indentations, 5 embryos (stage 13, basal); 20 indentations, 7 embryos (stage 15, apical); 55 indentations, 19 embryos (stage 15, basal). The apical surface (green) was softer than the basal surface (orange) at stage 13 (*p* = 4.2 × 10^−6^), but stiffer at stage 15 (*p* = 2.5 × 10^−7^). The box and whiskers indicate the maximum, third quartile, median, first quartile, and minimum values of each group; data beyond the end of the whiskers (the upper limit of whiskers is 1.5 times the box length) are shown as outliers; a cross mark indicates the mean value. *p*‐values were calculated using the two‐sided Wilcoxon rank‐sum test (***p < .001).

During NTC, the actomyosin activity level is preferentially elevated on the apical side of neural progenitor cells, which leads to apical constriction (Martin & Goldstein, 2014; Nandadasa et al., 2009; Nishimura & Takeichi, 2008; Sawyer et al., 2010; Suzuki et al., 2012; Suzuki et al., 2017). Thus, we hypothesized that the stiffness of the apical and basal surfaces of the neural plate would differ in magnitude. To test this hypothesis, we measured the elasticity of the apical and basal surfaces of neural‐plate explants in the presumptive anterior spinal cord region at stages 13 and 15 (Figure 2a, b). Similar to previous studies (Zhou et al., 2009; Zhou et al., 2010), we found clutch‐to‐clutch variation in the stiffness of tissue explants (data not shown). Therefore, in the analyses using tissue explants, we limited our focus on the difference in stiffness within the same clutch.

This analysis revealed a different balance of elasticity within the neural plates between stages 13 and 15. At stage 13, the apical surface was softer than the basal surface (*p* < 10^−5^; Figure 2c). In contrast, at stage 15, the apical surface was stiffer than the basal surface (*p* < 10^−6^; Figure 2d), indicating that a reversal of the stiffness relationship between the apical and basal surfaces was induced between stages 13 and 15. These results are consistent with an increase in apical‐specific actomyosin activity levels during neural plate bending (Nandadasa et al., 2009) and provide experimental support for our previously proposed hypothesis based on mathematical simulations that an increase in the elasticity of the apical side determines the initiation and direction of neural plate bending (Inoue et al., 2016; Suzuki et al., 2017).

### 3.3 Changes in mesoderm elasticity during the closure process

**FIGURE 3:**
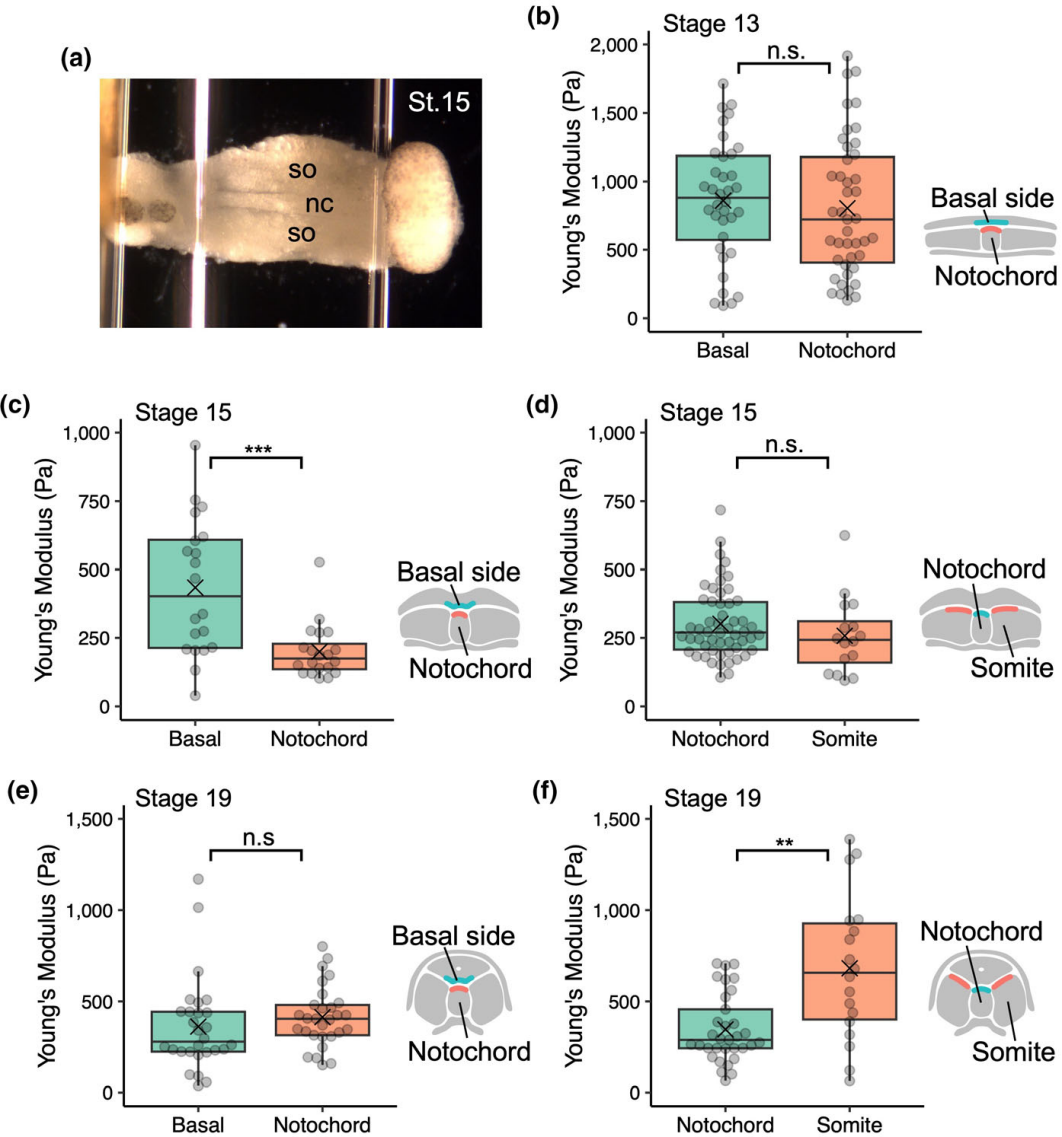
Elasticity of neural and mesodermal explants. (a) Representative atomic force microscopy image of mesodermal explant at stage 15. Anterior is to the left. nc, notochord; so, somites. (b, c, e) Stiffness of the basal surface of the neural plate and notochord at stages 13 (b), 15 (c), and 19 (e); *n* = 36 indentations, 4 embryos (stage 13, basal); 43 indentations, 5 embryos (stage 13, notochord); 20 indentations, 8 embryos (stage 15, basal); 20 indentations, 8 embryos (stage 15, notochord); 28 indentations, 5 embryos (stage 19, basal); 29 indentations, 4 embryos (stage 19, notochord). The basal surface of the neural plate (green) was softer than the notochord (orange) at stage 15 (*p* = 3.3 × 10^−4^) but not at stages 13 (*p* = .47) and 19 (*p* = .14). (d, f) Stiffness of the notochord and somite at stages 15 (d) and 19 (f); *n* = 54 indentations, 25 embryos (stage 15, notochord); 16 indentations, 11 embryos (stage 15, somite); 33 indentations, 5 embryos (stage 19, notochord); and 29 indentations, 4 embryos (stage 19, somite). The notochord (green) was softer than the somite (orange) at stage 19 (*p* = 1.3 × 10^−3^) but not at stage 15 (*p* = .19). The box and whiskers indicate the maximum, third quartile, median, first quartile, and minimum values of each group; data beyond the end of the whiskers (the upper limit of whiskers is 1.5 times the box length) are shown as outliers; a cross mark indicates the mean value. *p*‐values were calculated using the two‐sided Wilcoxon rank‐sum test (**p < .01; ***p < .001; n.s., not significant).

Our mathematical simulation also predicted that mechanical loading on the basal side of the neural plate would affect neural plate bending (Inoue et al., 2016). This prediction suggests that in addition to the neural plate itself, the stiffness of the mesoderm adjacent to the basal surface of the neural plate could affect NTC. Therefore, we measured the elasticity of the mesodermal explants and compared it with that of neural plate explants (Figure 3a).

At stage 13, the stiffness of the basal surface of the neural plate was not significantly different from that of the axial mesoderm, which was attached to the neural plate and underwent differentiation into a notochord (*p* = .47; Figure 3b). Intriguingly, at stage 15, the basal surface of the neural plate was stiffer than the notochord (*p* < 10^−3^; Figure 3c). However, the elasticity of somites under differentiation from the paraxial mesoderm did not differ from that of the notochord (*p* = .17; Figure 3d). Measurements at stage 19, when NTC was complete, revealed no significant difference between the elasticity of the basal surface of the neural tube and that of the notochord (*p* = .14; Figure 3e), whereas the somites exhibited greater elasticity than the notochord (*p* = .0013; Figure 3f). These results suggest that the mesoderm adjacent to the neural plate is softer than the neural plate during bending.

### 3.4 Delayed NTC by experimental activation of actomyosin in mesoderm

**FIGURE 4:**
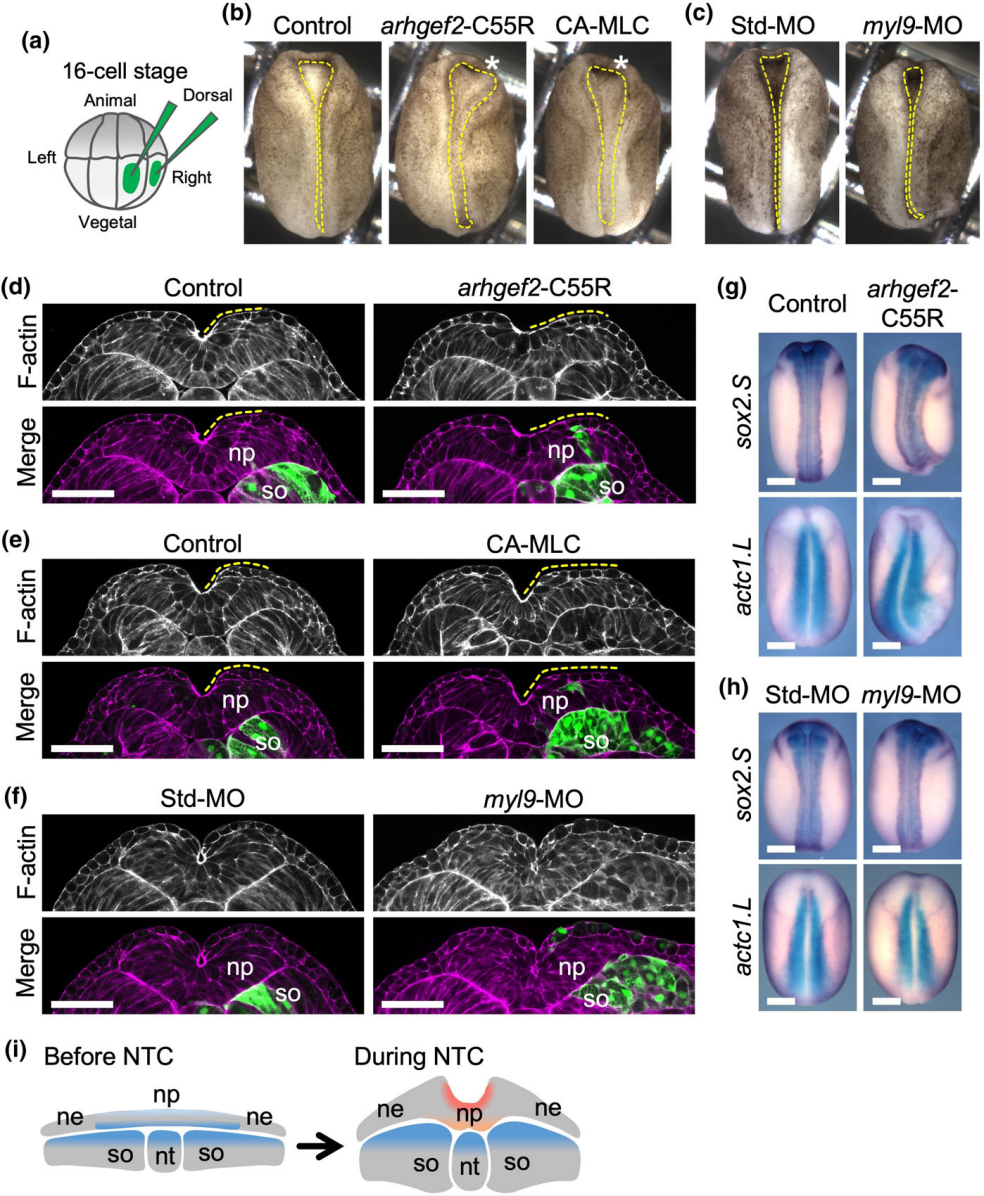
Experimental increase in mesoderm stiffness delays neural tube closure. (a) Schematic of microinjection. mRNAs or morpholino oligonucleotides (MOs) were injected into two right dorso‐vegetal blastomeres of 16‐cell‐stage embryos, which give rise to the notochord and somites. Enhanced green fluorescent protein (EGFP) mRNA was co‐injected as a tracer. (b, c) Dorsal views of unilaterally injected embryos at stages 16–17, anterior is to the top. Dashed lines indicate the outline of the neural plate. (b) Injection of *arhgef2*‐C55R or a constitutively active form of the myosin light chain (CA‐MLC) delayed the neural tube closure compared with the control; *n* = 0/23 (control); 17/19 (*arhgef2*‐C55R); 10/13 (CA‐MLC); *p*_*arhgef2*‐C55R_ = 2.0 × 10^−9^; *p*_CA‐MLC_ = 2.3 × 10^−6^). The asterisks indicate the opened neural tube on the injected side. (c) myosin light chain 9 (*myl9*)‐MO did not show an effect; *n* = 0/16 (Std‐MO); *n* = 1/20 (*myl9*‐MO); *p* = 1. (d–f) Phalloidin‐stained transverse sections through the neural plate at stages 16 (d, e) and 19 (f), unilaterally injected with *arhgef2*‐C55R (d), CA‐MLC (e), and *myl9*‐MO (f). The bottom panels show the merged images with EGFP (green). The dashed lines indicate the outline of the neural plate on the injected side. np, neural plate; so, somites. Scale bars: 100 μm. (g, h) In situ hybridization analysis of embryos injected with *arhgef2*‐C55R (g) and *myl9*‐MO (h). Dorsal views showing the expression of *sox2.S*, a pan‐neural marker (top), and *actc1.L*, a somite marker (bottom). Anterior is to the top. Expression patterns were similar between the manipulated and control embryos. *n* = 7 (control, *sox2.S*); *n* = 4 (*arhgef2*‐C55R, *sox2.S*); *n* = 7 (control, *actc1.L*); *n* = 4 (*arhgef2*‐C55R, *actc1.L*); *n* = 6 (Std‐MO, *sox2.S*); *n* = 6 (*myl9*‐MO, *sox2.S*); *n* = 7 (Std‐MO, *actc1.L*); *n* = 5 (*myl9*‐MO, *actc1.L*). Scale bars: 500 μm. *p*‐values were calculated using Fisher's exact test. *p*‐values for pairwise comparisons were adjusted using Holm's method. (i) A proposed model for the relationship of stiffness between the neural plate (np) and surrounding tissues in neural tube closure (NTC). Before NTC (left), the apical surface of the neural plate (light blue) is softer than the basal surface and mesodermal tissues (blue). During NTC (right), stiffness increases significantly at the apical surface of the neural plate (red). The basal surface (orange) shows intermediate stiffness between the apical surface and mesodermal tissues (blue).

To test the possible influence of mesoderm stiffness on NTC, we injected mRNA constructs that increased or decreased actomyosin activity levels unilaterally into the dorso‐vegetal blastomeres of 16‐cell stage embryos and examined the effect of mesoderm‐specific cell stiffness changes on NTC (Figure 4a).

Overexpression of the point‐mutant (C55R) of *arhgef2*, acting as a constitutively active RhoGEF and increasing the stiffness of the *Xenopus* mesoderm (Zhou et al., 2010), caused the neural tube on the injected side to remain open (*n* = 17/19, *p* < 10^−8^; Figure 4b, d) compared with the control (*n* = 0/23), suggesting a delay in NTC. Similar results were observed with overexpression of CA‐MLC (Barriga et al., 2018) (*n* = 10/13, *p* < 10^−5^; Figure 4b, e). The delay in NTC was also supported by the observation of phalloidin‐stained transverse sections with the widened neural plate where the constructs were introduced into somites on the right side (yellow dashed lines in Figure 4d, e). In contrast, no delay in NTC was observed following the knockdown of *myl9* by antisense morpholino (*n* = 1/20, *p* = 1; Figure 4c, f), which reduces tissue stiffness (Barriga et al., 2018; Shindo & Wallingford, 2014), compared with the control (*n* = 0/16). Expression patterns of the pan‐neural marker *sox2.S* and the somite marker *actc1.L*, as revealed by in situ hybridization, suggested that, although *arhgef2*‐C55R affected the shape of the somites, these constructs did not affect the differentiation of the neural ectoderm and paraxial mesoderm (Figure 4g, h).

## 4 CONCLUSION

Based on these results, we propose the following model for the stiffness relationship between the neural plate and surrounding tissues in NTC (Figure 4i), in which stiffness increases preferentially at the apical surface of the neural progenitor cells (Figure 1), thereby reversing the balance of elasticity such that the apical surface is stiffer than the basal surface (Figure 2). The trend of increased elasticity was limited to the neural plate, whereas no appreciable increase in elasticity was detected in the non‐neural ectoderm or adjacent mesoderm (Figures 1, 3). When cellular stiffness in the mesoderm was experimentally increased, a delay in NTC was induced (Figure 4). These results suggest that, in addition to tissue‐autonomous regulation, low stiffness in the surrounding tissues may be crucial for the successful progression of large‐scale morphogenetic movements such as NTC. Our previous mathematical simulations also support this idea (Inoue et al., 2016). However, in the mathematical model, the elasticity of the sheet lining the neural plate, which recapitulates the mechanical properties of the mesoderm, affects the closing movement when apicobasal cell elongation is inhibited. Our experimental results indicate that aberrant elevation of mesoderm stiffness affects NTC, even when cell elongation activity is normal in vivo. This suggests that NTC is more sensitive to changes in the stiffness of the mesoderm than that predicted by the simulation. Further elucidation of the mechanical and chemical mechanisms governing this sensitivity will provide insights into the mechanistic aspects of the pathogenesis of NTDs, one of the most frequent structural congenital abnormalities in humans.

## AUTHOR CONTRIBUTIONS

M.S. and N.U. designed the study. M.S. and N.Y. performed the experiments and analyzed the data. M.S. and N.U. wrote the paper with input from N.Y.

## Supporting information


**MOVIE S1.** Repeated indentations of the neural plate. Dorsal view of an embryo during repeated indentations of the neural plate; anterior is to the left. Elapsed time (min) is shown at the top right. A snapshot of this process is shown in Figure 1c.

**MOVIE S2.** Repeated indentations of the non‐neural ectoderm. Dorsolateral view of an embryo during repeated indentations of the non‐neural ectoderm; anterior is to the left. Elapsed time (min) is shown at the top right. A snapshot of this process is shown in Figure 1d.
